# {μ-2,2′-(Ethane-1,2-di­yl)bis­[4,6-bis­(tri­methyl­sil­yl)-1,3-di­hydro­cyclo­penta­[*c*]pyrrol-5-one]}bis­[tri­carbonyl­iron(0)]

**DOI:** 10.1107/S2414314623003462

**Published:** 2023-04-21

**Authors:** Hilario D. Huerta-Zerón, Anke Spannenberg, Matthias Beller, Henrik Junge

**Affiliations:** a Leibniz-Institut für Katalyse e. V., Albert-Einstein-Strasse 29a, 18059 Rostock, Germany; Benemérita Universidad Autónoma de Puebla, México

**Keywords:** crystal structure, iron carbonyl complex, cyclo­penta­dienone ligand, binuclear complex

## Abstract

The binuclear title compound, [Fe_2_(C_28_H_48_N_2_O_2_Si_4_)(CO)_6_], consists of two central iron(0) atoms, each of them surrounded by a cyclo­penta­dienone moiety and three carbonyl ligands in a three-legged piano-stool shape. Furthermore, the bis­(cyclo­penta­dienone) ligand acts as a bridge between the two metal atoms.

## Abstract 2

The binuclear title compound consists of two iron(0) centres, each of them surrounded by a cyclo­penta­dienone moiety and three carbonyl ligands in a three-legged piano-stool geometry.

## Graphical abstract

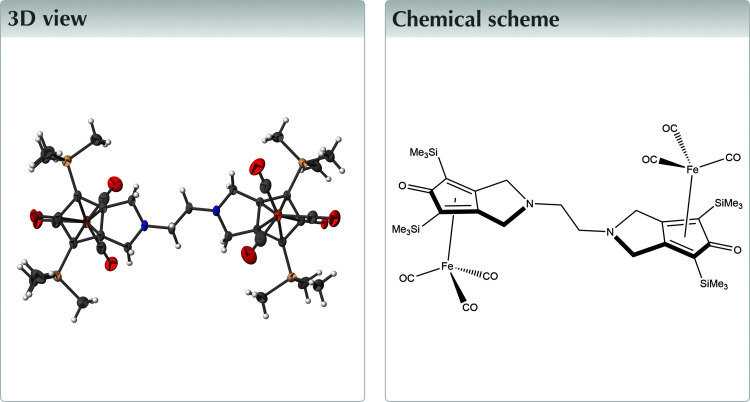

## Structure description

**Figure 1:**
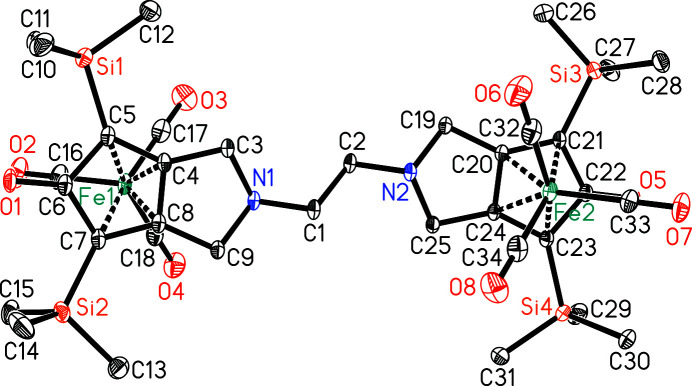
The mol­ecular structure of the title compound, showing the atom labelling and displacement ellipsoids drawn at 30% probability level. H atoms have been omitted for clarity.

**Figure 2:**
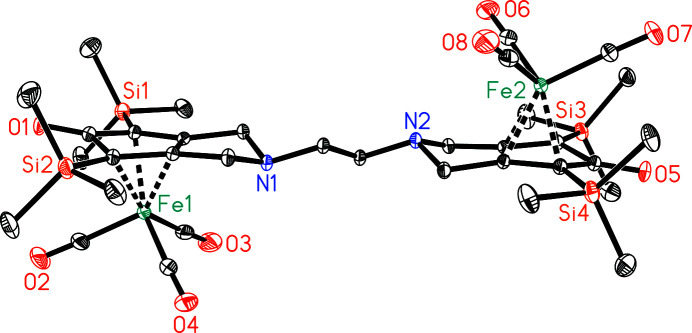
Side view of the title compound. H atoms have been omitted for clarity. Displacement ellipsoids are drawn at the 30% probability level.

The title compound is a binuclear complex where both iron(0) atoms exhibit a piano-stool coordination environment. Each iron(0) atom is surrounded by a cyclo­penta­dienone moiety in a η^4^-coordination mode [torsion angles C5—C4—C8—C7 = −0.7 (2)° and C21—C20—C24—C23 = 0.0 (2)°] and three carbonyl ligands (Fig. 1 ▸). The distances between Fe1 and atoms C4, C5, C7 and C8 range from 2.0698 (19) to 2.154 (2) Å [for Fe2 and atoms C20, C21, C23 and C24, the corresponding range is 2.0702 (18)–2.1435 (19) Å], while the Fe1—C6 distance is 2.3591 (15) Å [Fe2—C22 = 2.3497 (18) Å]. The pyrrolidine N atoms are 0.49 Å out of the planes defined by atoms C3/C4/C8/C9 and C19/C20/C24/C25, respectively, resulting in an envelope conformation for both heterocycles. The Fe(CO)_3_ units are located on opposite sides of the bis­(cyclo­penta­dienone) ligand, which bridges both metal atoms (Fig. 2 ▸). The observed η^4^-coordination mode is in agreement with several reported iron(0) cyclo­penta­dienone tricarbonyl complexes [see, for example, Knölker *et al.* (1992 ▸) and Hackl *et al.* (2022 ▸)].

## Synthesis and crystallization

The iron precursor Fe_2_(CO)_9_ (471 mg, 1.29 mmol) was weighted and transferred to a 50 ml Schlenk tube equipped with a stirring bar in a glove-box. Next, the tetra­yne *N* ^1^,*N* ^1^,*N* ^2^,*N* ^2^-tetra­kis­[3-(tri­methyl­sil­yl)prop-2-yn-1-yl]ethane-1,2-di­amine (321 mg, 0.64 mmol) was dissolved in 13 ml of dry toluene in another flask. The tetra­yne solution and the iron precursor were then mixed in the Schlenk tube and heated to 383 K for 16 h. The resulting black mixture was filtered through Celite. The filtrate was further purified *via* column chromatography over silica gel with penta­ne/ethyl acetate (90:10 *v*/*v*) as eluent to finally afford a brown solid (yield 0.497 g, 93%). Crystals suitable for X-ray analysis were obtained by slow diffusion of pentane into a solution of the complex in di­chloro­methane. ^1^H NMR (300 MHz, CDCl_3_): δ (ppm) 3.92 (*d*, *J* = 12.9 Hz, 4H), 3.42 (*d*, *J* = 13.0 Hz, 4H), 3.06 (*s*, 4H), 0.27 (*s*, 36H). ^13^C NMR (100 MHz, CDCl_3_): δ (ppm) 208.69 (C≡O), 182.39 (C=O), 113.06 (C_Cp_), 69.33 (C_Cp_), 54.36 (–CH_2_—CH_2_–), 53.72 (C_pyr_—N), −0.69 (SiMe_3_). HR–MS (ESI): theoretical mass for C_34_H_48_Fe_2_N_2_O_8_Si_4_: 836.11811; found: 836.11579.

## Refinement

Crystal data, data collection and structure refinement details are summarized in Table 1 ▸.

## Supplementary Material

Crystal structure: contains datablock(s) I, global. DOI: 10.1107/S2414314623003462/bh4073sup1.cif

Structure factors: contains datablock(s) I. DOI: 10.1107/S2414314623003462/bh4073Isup2.hkl

CCDC reference: 2256807

Additional supporting information:  crystallographic information; 3D view; checkCIF report

## Figures and Tables

**Table 1:** Experimental details

Crystal data	
Chemical formula	[Fe_2_(C_28_H_48_N_2_O_2_Si_4_)(CO)_6_]
*M*_r_	836.80
Crystal system, space group	Monoclinic, *P*2_1_/*n*
Temperature (K)	150
*a*, *b*, *c* (Å)	17.8815 (13), 12.5896 (10), 20.3917 (16)
β (°)	112.0104 (16)
*V* (Å^3^)	4256.0 (6)
*Z*	4
Radiation type	Mo *K*α
μ (mm^−1^)	0.84
Crystal size (mm)	0.18 × 0.17 × 0.04

Data collection	
Diffractometer	Bruker APEXII CCD
Absorption correction	Multi-scan (*SADABS*; Bruker, 2014 ▸)
*T*_min_, *T*_max_	0.86, 0.97
No. of measured, independent and observed [*I* > 2σ(*I*)] reflections	57670, 11074, 7588
*R*_int_	0.048
(sin θ/λ)_max_ (Å^−1^)	0.677

Refinement	
*R*[*F* ^2^ > 2σ(*F* ^2^)], *wR*(*F* ^2^), *S*	0.038, 0.096, 1.00
No. of reflections	11074
No. of parameters	463
H-atom treatment	H-atom parameters constrained
Δρ_max_, Δρ_min_ (e Å^−3^)	0.47, −0.27

Computer programs: *APEX2* (Bruker, 2014 ▸), *SAINT* (Bruker, 2013 ▸), *SHELXS97* (Sheldrick, 2008 ▸), *SHELXL2018* (Sheldrick, 2015 ▸), *XP* in *SHELXTL* (Sheldrick, 2008 ▸) and *publCIF* (Westrip, 2010 ▸).

## full crystallographic data

### Crystal data

**Table:**

[Fe_2_(C_28_H_48_N_2_O_2_Si_4_)(CO)_6_]	*F*(000) = 1752
*M**_r_* = 836.80	*D*_x_ = 1.306 Mg m^−^^3^
Monoclinic, *P*2_1_/*n*	Mo *K*α radiation, λ = 0.71073 Å
*a* = 17.8815 (13) Å	Cell parameters from 9955 reflections
*b* = 12.5896 (10) Å	θ = 2.5–28.2°
*c* = 20.3917 (16) Å	µ = 0.84 mm^−^^1^
β = 112.0104 (16)°	*T* = 150 K
*V* = 4256.0 (6) Å^3^	Plate, pale yellow
*Z* = 4	0.18 × 0.17 × 0.04 mm

### Data collection

**Table:**

Bruker APEXII CCD diffractometer	11074 independent reflections
Radiation source: fine-focus sealed tube	7588 reflections with *I* > 2σ(*I*)
Detector resolution: 8.3333 pixels mm^-1^	*R*_int_ = 0.048
φ and ω scans	θ_max_ = 28.8°, θ_min_ = 1.3°
Absorption correction: multi-scan (SADABS; Bruker, 2014)	*h* = −24→24
*T*_min_ = 0.86, *T*_max_ = 0.97	*k* = −17→16
57670 measured reflections	*l* = −27→27

### Refinement

**Table:**

Refinement on *F*^2^	Primary atom site location: structure-invariant direct methods
Least-squares matrix: full	Secondary atom site location: difference Fourier map
*R*[*F*^2^ > 2σ(*F*^2^)] = 0.038	Hydrogen site location: inferred from neighbouring sites
*wR*(*F*^2^) = 0.096	H-atom parameters constrained
*S* = 1.00	*w* = 1/[σ^2^(*F*_o_^2^) + (0.0438*P*)^2^ + 1.078*P*] where *P* = (*F*_o_^2^ + 2*F*_c_^2^)/3
11074 reflections	(Δ/σ)_max_ = 0.002
463 parameters	Δρ_max_ = 0.47 e Å^−^^3^
0 restraints	Δρ_min_ = −0.27 e Å^−^^3^
0 constraints	

### Fractional atomic coordinates and isotropic or equivalent isotropic displacement parameters (Å^2^)

**Table:**

	*x*	*y*	*z*	*U*_iso_*/*U*_eq_	
C1	0.54321 (11)	0.63613 (14)	0.28191 (10)	0.0253 (4)	
H1A	0.542523	0.627454	0.329938	0.030*	
H1B	0.580562	0.582548	0.275743	0.030*	
C2	0.45943 (11)	0.61771 (14)	0.22752 (10)	0.0253 (4)	
H2A	0.421846	0.670743	0.233925	0.030*	
H2B	0.459901	0.626777	0.179456	0.030*	
C3	0.58418 (12)	0.76032 (14)	0.20761 (10)	0.0239 (4)	
H3A	0.533264	0.781359	0.169093	0.029*	
H3B	0.605961	0.696022	0.193141	0.029*	
C4	0.64447 (11)	0.84938 (14)	0.22697 (10)	0.0229 (4)	
C5	0.67853 (12)	0.92754 (14)	0.19534 (10)	0.0256 (4)	
C6	0.75207 (12)	0.96754 (15)	0.25395 (10)	0.0282 (4)	
C7	0.74542 (12)	0.93201 (15)	0.32135 (10)	0.0275 (4)	
C8	0.68358 (12)	0.85252 (14)	0.30142 (10)	0.0242 (4)	
C9	0.64977 (12)	0.76639 (15)	0.33303 (10)	0.0262 (4)	
H9A	0.685394	0.703279	0.345309	0.031*	
H9B	0.641462	0.791611	0.375784	0.031*	
C10	0.75004 (15)	0.9193 (2)	0.08299 (13)	0.0489 (6)	
H10A	0.743478	0.940491	0.034901	0.073*	
H10B	0.756785	0.842055	0.087736	0.073*	
H10C	0.797757	0.954243	0.117079	0.073*	
C11	0.64146 (15)	1.10465 (17)	0.08631 (13)	0.0458 (6)	
H11A	0.637865	1.123095	0.038534	0.069*	
H11B	0.686335	1.143475	0.121156	0.069*	
H11C	0.590981	1.123916	0.091725	0.069*	
C12	0.56817 (14)	0.88456 (17)	0.04283 (12)	0.0408 (5)	
H12A	0.521104	0.907238	0.052845	0.061*	
H12B	0.577001	0.808302	0.052165	0.061*	
H12C	0.558696	0.898826	−0.006898	0.061*	
C13	0.80294 (17)	0.89301 (18)	0.47992 (12)	0.0478 (6)	
H13A	0.844560	0.909761	0.526121	0.072*	
H13B	0.802238	0.816259	0.471681	0.072*	
H13C	0.750160	0.915638	0.479101	0.072*	
C14	0.92329 (15)	0.9168 (2)	0.40640 (14)	0.0595 (8)	
H14A	0.934169	0.954216	0.368779	0.089*	
H14B	0.920101	0.840232	0.396958	0.089*	
H14C	0.966896	0.931015	0.451963	0.089*	
C15	0.82806 (18)	1.10948 (18)	0.42381 (13)	0.0529 (7)	
H15A	0.841907	1.145488	0.387263	0.079*	
H15B	0.868657	1.126231	0.470517	0.079*	
H15C	0.774979	1.133635	0.421347	0.079*	
C16	0.65391 (16)	1.13489 (17)	0.26192 (13)	0.0435 (6)	
C17	0.52601 (16)	0.99906 (17)	0.20060 (13)	0.0396 (5)	
C18	0.59376 (14)	1.00138 (17)	0.33755 (12)	0.0378 (5)	
C19	0.35354 (12)	0.48435 (14)	0.17820 (10)	0.0238 (4)	
H19A	0.360733	0.460784	0.134617	0.029*	
H19B	0.316187	0.545683	0.166999	0.029*	
C20	0.32403 (11)	0.39541 (13)	0.21124 (9)	0.0212 (4)	
C21	0.26507 (11)	0.31285 (14)	0.19311 (10)	0.0228 (4)	
C22	0.26143 (11)	0.27612 (15)	0.26133 (10)	0.0239 (4)	
C23	0.33460 (11)	0.31949 (14)	0.31844 (10)	0.0236 (4)	
C24	0.36530 (11)	0.39952 (14)	0.28545 (9)	0.0216 (4)	
C25	0.42301 (12)	0.49104 (14)	0.30329 (10)	0.0240 (4)	
H25A	0.400448	0.553799	0.318663	0.029*	
H25B	0.475312	0.471854	0.340702	0.029*	
C26	0.20472 (17)	0.3520 (2)	0.03474 (11)	0.0503 (7)	
H26A	0.165123	0.330968	−0.011509	0.075*	
H26B	0.259212	0.336653	0.036604	0.075*	
H26C	0.199645	0.428263	0.041826	0.075*	
C27	0.08705 (14)	0.31461 (19)	0.10881 (13)	0.0452 (6)	
H27A	0.077609	0.273686	0.145860	0.068*	
H27B	0.044058	0.299651	0.063014	0.068*	
H27C	0.087316	0.390608	0.119287	0.068*	
C28	0.19327 (15)	0.13189 (17)	0.08980 (12)	0.0401 (5)	
H28A	0.188460	0.091525	0.129123	0.060*	
H28B	0.245440	0.116581	0.086417	0.060*	
H28C	0.149680	0.111422	0.045567	0.060*	
C29	0.26805 (16)	0.3313 (2)	0.43367 (13)	0.0481 (6)	
H29A	0.221455	0.288209	0.405390	0.072*	
H29B	0.256378	0.406449	0.421768	0.072*	
H29C	0.279097	0.320524	0.484041	0.072*	
C30	0.37827 (15)	0.14624 (16)	0.43158 (12)	0.0438 (6)	
H30A	0.425809	0.126390	0.421394	0.066*	
H30B	0.331540	0.105284	0.401166	0.066*	
H30C	0.388250	0.131041	0.481289	0.066*	
C31	0.44746 (15)	0.37099 (18)	0.46791 (12)	0.0459 (6)	
H31A	0.458876	0.359929	0.518313	0.069*	
H31B	0.436422	0.446385	0.456374	0.069*	
H31C	0.494221	0.348912	0.457217	0.069*	
C32	0.41297 (14)	0.24965 (17)	0.17171 (13)	0.0397 (5)	
C33	0.36184 (14)	0.11454 (16)	0.25217 (12)	0.0361 (5)	
C34	0.48747 (15)	0.25614 (17)	0.30877 (14)	0.0403 (5)	
Fe1	0.62688 (2)	0.99611 (2)	0.26491 (2)	0.02856 (8)	
Fe2	0.38508 (2)	0.25415 (2)	0.24730 (2)	0.02577 (8)	
N1	0.57218 (10)	0.74294 (12)	0.27495 (8)	0.0240 (3)	
N2	0.43155 (9)	0.51043 (12)	0.23474 (8)	0.0236 (3)	
O1	0.80358 (9)	1.02818 (12)	0.24807 (8)	0.0372 (4)	
O2	0.67270 (13)	1.22071 (13)	0.25975 (11)	0.0636 (6)	
O3	0.46212 (12)	0.99708 (15)	0.15841 (11)	0.0586 (5)	
O4	0.57245 (12)	1.00074 (14)	0.38359 (10)	0.0546 (5)	
O5	0.21195 (8)	0.21317 (11)	0.26877 (7)	0.0316 (3)	
O6	0.42889 (13)	0.24959 (14)	0.12265 (10)	0.0624 (5)	
O7	0.34550 (12)	0.02768 (12)	0.25565 (10)	0.0543 (5)	
O8	0.55219 (11)	0.26059 (15)	0.34928 (11)	0.0619 (5)	
Si1	0.65890 (4)	0.95934 (4)	0.10059 (3)	0.03152 (13)	
Si2	0.82551 (4)	0.96410 (5)	0.40924 (3)	0.03588 (15)	
Si3	0.18610 (4)	0.27641 (4)	0.10551 (3)	0.02961 (13)	
Si4	0.35781 (4)	0.29061 (4)	0.41428 (3)	0.03062 (14)	

### Atomic displacement parameters (Å^2^)

**Table:**

	*U*^11^	*U*^22^	*U*^33^	*U*^12^	*U*^13^	*U*^23^
C1	0.0221 (10)	0.0188 (9)	0.0380 (11)	−0.0034 (8)	0.0148 (9)	0.0011 (7)
C2	0.0235 (10)	0.0190 (9)	0.0367 (10)	−0.0038 (8)	0.0150 (9)	0.0021 (7)
C3	0.0226 (10)	0.0187 (8)	0.0320 (10)	−0.0043 (8)	0.0121 (8)	−0.0009 (7)
C4	0.0196 (10)	0.0182 (8)	0.0341 (10)	−0.0004 (7)	0.0137 (8)	−0.0021 (7)
C5	0.0252 (11)	0.0209 (9)	0.0357 (10)	−0.0030 (8)	0.0170 (9)	−0.0015 (7)
C6	0.0290 (11)	0.0238 (10)	0.0386 (11)	−0.0050 (8)	0.0205 (10)	−0.0045 (8)
C7	0.0273 (11)	0.0245 (9)	0.0356 (10)	−0.0066 (8)	0.0175 (9)	−0.0058 (8)
C8	0.0245 (10)	0.0195 (8)	0.0335 (10)	−0.0025 (8)	0.0166 (9)	−0.0026 (7)
C9	0.0256 (11)	0.0240 (9)	0.0309 (10)	−0.0060 (8)	0.0127 (9)	−0.0004 (7)
C10	0.0511 (16)	0.0623 (16)	0.0440 (13)	−0.0006 (13)	0.0303 (13)	0.0026 (12)
C11	0.0479 (16)	0.0313 (12)	0.0519 (14)	−0.0094 (11)	0.0113 (12)	0.0089 (10)
C12	0.0448 (15)	0.0298 (11)	0.0404 (12)	0.0012 (10)	0.0073 (11)	0.0016 (9)
C13	0.0707 (19)	0.0366 (12)	0.0381 (12)	−0.0142 (12)	0.0229 (13)	−0.0070 (10)
C14	0.0347 (15)	0.084 (2)	0.0537 (16)	−0.0127 (14)	0.0096 (13)	−0.0220 (15)
C15	0.084 (2)	0.0388 (13)	0.0467 (14)	−0.0325 (13)	0.0372 (14)	−0.0178 (10)
C16	0.0567 (16)	0.0245 (11)	0.0629 (16)	−0.0025 (11)	0.0380 (14)	−0.0038 (10)
C17	0.0430 (15)	0.0276 (11)	0.0574 (14)	0.0064 (10)	0.0294 (13)	0.0000 (10)
C18	0.0413 (14)	0.0277 (10)	0.0519 (13)	−0.0031 (10)	0.0261 (12)	−0.0062 (9)
C19	0.0256 (11)	0.0193 (8)	0.0284 (9)	−0.0039 (8)	0.0123 (8)	0.0000 (7)
C20	0.0211 (10)	0.0177 (8)	0.0270 (9)	0.0001 (7)	0.0113 (8)	0.0005 (7)
C21	0.0219 (10)	0.0187 (8)	0.0285 (9)	−0.0031 (7)	0.0102 (8)	0.0000 (7)
C22	0.0216 (10)	0.0218 (9)	0.0304 (10)	−0.0020 (7)	0.0121 (9)	0.0007 (7)
C23	0.0214 (10)	0.0218 (9)	0.0285 (9)	−0.0011 (8)	0.0105 (8)	0.0018 (7)
C24	0.0183 (10)	0.0188 (8)	0.0288 (9)	−0.0008 (7)	0.0101 (8)	0.0013 (7)
C25	0.0227 (10)	0.0209 (9)	0.0283 (9)	−0.0035 (8)	0.0096 (8)	0.0014 (7)
C26	0.0654 (18)	0.0487 (14)	0.0306 (12)	−0.0167 (13)	0.0109 (12)	0.0034 (10)
C27	0.0317 (13)	0.0446 (13)	0.0478 (13)	−0.0022 (11)	0.0018 (11)	−0.0095 (11)
C28	0.0470 (15)	0.0324 (11)	0.0424 (12)	−0.0122 (10)	0.0184 (11)	−0.0111 (9)
C29	0.0637 (18)	0.0480 (14)	0.0440 (13)	−0.0034 (13)	0.0332 (13)	0.0041 (11)
C30	0.0545 (16)	0.0286 (11)	0.0400 (12)	−0.0059 (11)	0.0080 (11)	0.0116 (9)
C31	0.0520 (16)	0.0346 (12)	0.0353 (12)	−0.0099 (11)	−0.0018 (11)	0.0037 (9)
C32	0.0446 (14)	0.0281 (11)	0.0566 (14)	0.0037 (10)	0.0306 (12)	0.0001 (10)
C33	0.0399 (13)	0.0261 (11)	0.0459 (12)	0.0016 (9)	0.0200 (11)	0.0038 (9)
C34	0.0336 (14)	0.0301 (11)	0.0593 (15)	0.0060 (10)	0.0200 (12)	0.0011 (10)
Fe1	0.03200 (18)	0.01828 (13)	0.04369 (17)	−0.00303 (12)	0.02367 (14)	−0.00285 (11)
Fe2	0.02530 (16)	0.01803 (13)	0.03728 (16)	−0.00013 (11)	0.01553 (13)	0.00141 (11)
N1	0.0226 (9)	0.0189 (7)	0.0337 (8)	−0.0052 (7)	0.0144 (7)	0.0000 (6)
N2	0.0219 (9)	0.0198 (7)	0.0308 (8)	−0.0045 (6)	0.0119 (7)	0.0008 (6)
O1	0.0361 (9)	0.0380 (8)	0.0461 (9)	−0.0187 (7)	0.0252 (7)	−0.0073 (7)
O2	0.0911 (16)	0.0238 (9)	0.0954 (15)	−0.0126 (9)	0.0573 (13)	−0.0054 (8)
O3	0.0398 (11)	0.0579 (12)	0.0734 (13)	0.0154 (9)	0.0158 (10)	−0.0028 (10)
O4	0.0680 (13)	0.0533 (11)	0.0628 (11)	−0.0043 (9)	0.0477 (11)	−0.0100 (9)
O5	0.0271 (8)	0.0323 (7)	0.0370 (8)	−0.0119 (6)	0.0138 (7)	0.0033 (6)
O6	0.0900 (16)	0.0528 (11)	0.0730 (13)	0.0060 (10)	0.0633 (12)	0.0004 (9)
O7	0.0717 (14)	0.0236 (8)	0.0732 (12)	−0.0039 (8)	0.0335 (11)	0.0048 (8)
O8	0.0292 (10)	0.0589 (12)	0.0848 (14)	0.0089 (9)	0.0068 (10)	−0.0031 (10)
Si1	0.0348 (3)	0.0280 (3)	0.0339 (3)	−0.0022 (3)	0.0154 (3)	0.0038 (2)
Si2	0.0408 (4)	0.0352 (3)	0.0345 (3)	−0.0163 (3)	0.0174 (3)	−0.0126 (2)
Si3	0.0327 (3)	0.0258 (3)	0.0275 (3)	−0.0085 (2)	0.0081 (2)	−0.0032 (2)
Si4	0.0376 (4)	0.0256 (3)	0.0261 (3)	−0.0057 (2)	0.0090 (3)	0.0049 (2)

### Geometric parameters (Å, º)

**Table:**

C1—N1	1.467 (2)	C17—Fe1	1.789 (3)
C1—C2	1.511 (3)	C18—O4	1.138 (3)
C1—H1A	0.9900	C18—Fe1	1.791 (2)
C1—H1B	0.9900	C19—N2	1.476 (2)
C2—N2	1.466 (2)	C19—C20	1.501 (2)
C2—H2A	0.9900	C19—H19A	0.9900
C2—H2B	0.9900	C19—H19B	0.9900
C3—N1	1.483 (2)	C20—C24	1.414 (3)
C3—C4	1.502 (2)	C20—C21	1.427 (2)
C3—H3A	0.9900	C20—Fe2	2.0721 (18)
C3—H3B	0.9900	C21—C22	1.490 (2)
C4—C8	1.414 (3)	C21—Si3	1.8721 (19)
C4—C5	1.432 (2)	C21—Fe2	2.1435 (19)
C4—Fe1	2.0719 (17)	C22—O5	1.239 (2)
C5—C6	1.494 (3)	C22—C23	1.491 (3)
C5—Si1	1.873 (2)	C22—Fe2	2.3497 (18)
C5—Fe1	2.1431 (18)	C23—C24	1.430 (2)
C6—O1	1.236 (2)	C23—Si4	1.8750 (19)
C6—C7	1.492 (3)	C23—Fe2	2.1394 (18)
C6—Fe1	2.3591 (19)	C24—C25	1.498 (2)
C7—C8	1.433 (3)	C24—Fe2	2.0702 (18)
C7—Si2	1.872 (2)	C25—N2	1.482 (2)
C7—Fe1	2.154 (2)	C25—H25A	0.9900
C8—C9	1.500 (2)	C25—H25B	0.9900
C8—Fe1	2.0698 (19)	C26—Si3	1.860 (2)
C9—N1	1.478 (2)	C26—H26A	0.9800
C9—H9A	0.9900	C26—H26B	0.9800
C9—H9B	0.9900	C26—H26C	0.9800
C10—Si1	1.865 (2)	C27—Si3	1.861 (2)
C10—H10A	0.9800	C27—H27A	0.9800
C10—H10B	0.9800	C27—H27B	0.9800
C10—H10C	0.9800	C27—H27C	0.9800
C11—Si1	1.860 (2)	C28—Si3	1.860 (2)
C11—H11A	0.9800	C28—H28A	0.9800
C11—H11B	0.9800	C28—H28B	0.9800
C11—H11C	0.9800	C28—H28C	0.9800
C12—Si1	1.864 (2)	C29—Si4	1.863 (2)
C12—H12A	0.9800	C29—H29A	0.9800
C12—H12B	0.9800	C29—H29B	0.9800
C12—H12C	0.9800	C29—H29C	0.9800
C13—Si2	1.865 (2)	C30—Si4	1.862 (2)
C13—H13A	0.9800	C30—H30A	0.9800
C13—H13B	0.9800	C30—H30B	0.9800
C13—H13C	0.9800	C30—H30C	0.9800
C14—Si2	1.869 (3)	C31—Si4	1.864 (2)
C14—H14A	0.9800	C31—H31A	0.9800
C14—H14B	0.9800	C31—H31B	0.9800
C14—H14C	0.9800	C31—H31C	0.9800
C15—Si2	1.852 (2)	C32—O6	1.138 (3)
C15—H15A	0.9800	C32—Fe2	1.790 (2)
C15—H15B	0.9800	C33—O7	1.141 (2)
C15—H15C	0.9800	C33—Fe2	1.817 (2)
C16—O2	1.137 (3)	C34—O8	1.144 (3)
C16—Fe1	1.820 (2)	C34—Fe2	1.790 (3)
C17—O3	1.144 (3)		
			
N1—C1—C2	111.02 (15)	N2—C25—H25A	111.4
N1—C1—H1A	109.4	C24—C25—H25A	111.4
C2—C1—H1A	109.4	N2—C25—H25B	111.4
N1—C1—H1B	109.4	C24—C25—H25B	111.4
C2—C1—H1B	109.4	H25A—C25—H25B	109.3
H1A—C1—H1B	108.0	Si3—C26—H26A	109.5
N2—C2—C1	110.40 (15)	Si3—C26—H26B	109.5
N2—C2—H2A	109.6	H26A—C26—H26B	109.5
C1—C2—H2A	109.6	Si3—C26—H26C	109.5
N2—C2—H2B	109.6	H26A—C26—H26C	109.5
C1—C2—H2B	109.6	H26B—C26—H26C	109.5
H2A—C2—H2B	108.1	Si3—C27—H27A	109.5
N1—C3—C4	101.86 (14)	Si3—C27—H27B	109.5
N1—C3—H3A	111.4	H27A—C27—H27B	109.5
C4—C3—H3A	111.4	Si3—C27—H27C	109.5
N1—C3—H3B	111.4	H27A—C27—H27C	109.5
C4—C3—H3B	111.4	H27B—C27—H27C	109.5
H3A—C3—H3B	109.3	Si3—C28—H28A	109.5
C8—C4—C5	109.99 (16)	Si3—C28—H28B	109.5
C8—C4—C3	108.69 (15)	H28A—C28—H28B	109.5
C5—C4—C3	141.19 (17)	Si3—C28—H28C	109.5
C8—C4—Fe1	69.96 (10)	H28A—C28—H28C	109.5
C5—C4—Fe1	72.85 (10)	H28B—C28—H28C	109.5
C3—C4—Fe1	124.69 (13)	Si4—C29—H29A	109.5
C4—C5—C6	105.47 (16)	Si4—C29—H29B	109.5
C4—C5—Si1	131.67 (15)	H29A—C29—H29B	109.5
C6—C5—Si1	121.43 (13)	Si4—C29—H29C	109.5
C4—C5—Fe1	67.48 (10)	H29A—C29—H29C	109.5
C6—C5—Fe1	78.65 (11)	H29B—C29—H29C	109.5
Si1—C5—Fe1	129.51 (10)	Si4—C30—H30A	109.5
O1—C6—C7	126.44 (18)	Si4—C30—H30B	109.5
O1—C6—C5	126.50 (18)	H30A—C30—H30B	109.5
C7—C6—C5	106.60 (15)	Si4—C30—H30C	109.5
O1—C6—Fe1	133.07 (15)	H30A—C30—H30C	109.5
C7—C6—Fe1	63.40 (10)	H30B—C30—H30C	109.5
C5—C6—Fe1	62.96 (10)	Si4—C31—H31A	109.5
C8—C7—C6	105.53 (16)	Si4—C31—H31B	109.5
C8—C7—Si2	130.90 (15)	H31A—C31—H31B	109.5
C6—C7—Si2	121.39 (14)	Si4—C31—H31C	109.5
C8—C7—Fe1	67.03 (11)	H31A—C31—H31C	109.5
C6—C7—Fe1	78.33 (12)	H31B—C31—H31C	109.5
Si2—C7—Fe1	132.64 (10)	O6—C32—Fe2	177.6 (2)
C4—C8—C7	110.03 (16)	O7—C33—Fe2	178.1 (2)
C4—C8—C9	108.93 (16)	O8—C34—Fe2	177.4 (2)
C7—C8—C9	140.88 (18)	C17—Fe1—C18	92.89 (11)
C4—C8—Fe1	70.11 (10)	C17—Fe1—C16	99.37 (11)
C7—C8—Fe1	73.37 (11)	C18—Fe1—C16	99.58 (10)
C9—C8—Fe1	124.35 (13)	C17—Fe1—C8	120.34 (9)
N1—C9—C8	102.04 (15)	C18—Fe1—C8	90.23 (9)
N1—C9—H9A	111.4	C16—Fe1—C8	138.60 (10)
C8—C9—H9A	111.4	C17—Fe1—C4	89.84 (9)
N1—C9—H9B	111.4	C18—Fe1—C4	119.05 (8)
C8—C9—H9B	111.4	C16—Fe1—C4	139.83 (9)
H9A—C9—H9B	109.2	C8—Fe1—C4	39.93 (7)
Si1—C10—H10A	109.5	C17—Fe1—C5	95.12 (9)
Si1—C10—H10B	109.5	C18—Fe1—C5	157.02 (9)
H10A—C10—H10B	109.5	C16—Fe1—C5	100.30 (9)
Si1—C10—H10C	109.5	C8—Fe1—C5	67.16 (7)
H10A—C10—H10C	109.5	C4—Fe1—C5	39.66 (7)
H10B—C10—H10C	109.5	C17—Fe1—C7	156.80 (8)
Si1—C11—H11A	109.5	C18—Fe1—C7	97.83 (9)
Si1—C11—H11B	109.5	C16—Fe1—C7	99.04 (10)
H11A—C11—H11B	109.5	C8—Fe1—C7	39.59 (7)
Si1—C11—H11C	109.5	C4—Fe1—C7	66.97 (7)
H11A—C11—H11C	109.5	C5—Fe1—C7	67.72 (7)
H11B—C11—H11C	109.5	C17—Fe1—C6	131.71 (9)
Si1—C12—H12A	109.5	C18—Fe1—C6	134.73 (9)
Si1—C12—H12B	109.5	C16—Fe1—C6	82.52 (9)
H12A—C12—H12B	109.5	C8—Fe1—C6	63.05 (7)
Si1—C12—H12C	109.5	C4—Fe1—C6	63.03 (7)
H12A—C12—H12C	109.5	C5—Fe1—C6	38.40 (7)
H12B—C12—H12C	109.5	C7—Fe1—C6	38.27 (7)
Si2—C13—H13A	109.5	C34—Fe2—C32	93.53 (11)
Si2—C13—H13B	109.5	C34—Fe2—C33	99.79 (10)
H13A—C13—H13B	109.5	C32—Fe2—C33	99.18 (10)
Si2—C13—H13C	109.5	C34—Fe2—C24	89.91 (9)
H13A—C13—H13C	109.5	C32—Fe2—C24	119.57 (8)
H13B—C13—H13C	109.5	C33—Fe2—C24	139.44 (9)
Si2—C14—H14A	109.5	C34—Fe2—C20	120.07 (9)
Si2—C14—H14B	109.5	C32—Fe2—C20	90.07 (9)
H14A—C14—H14B	109.5	C33—Fe2—C20	138.48 (9)
Si2—C14—H14C	109.5	C24—Fe2—C20	39.93 (7)
H14A—C14—H14C	109.5	C34—Fe2—C23	95.77 (9)
H14B—C14—H14C	109.5	C32—Fe2—C23	157.01 (8)
Si2—C15—H15A	109.5	C33—Fe2—C23	99.89 (9)
Si2—C15—H15B	109.5	C24—Fe2—C23	39.68 (7)
H15A—C15—H15B	109.5	C20—Fe2—C23	67.09 (7)
Si2—C15—H15C	109.5	C34—Fe2—C21	156.91 (8)
H15A—C15—H15C	109.5	C32—Fe2—C21	96.61 (9)
H15B—C15—H15C	109.5	C33—Fe2—C21	99.00 (9)
O2—C16—Fe1	178.1 (2)	C24—Fe2—C21	67.04 (7)
O3—C17—Fe1	177.2 (2)	C20—Fe2—C21	39.53 (7)
O4—C18—Fe1	177.5 (2)	C23—Fe2—C21	67.78 (7)
N2—C19—C20	101.75 (14)	C34—Fe2—C22	132.48 (9)
N2—C19—H19A	111.4	C32—Fe2—C22	133.40 (9)
C20—C19—H19A	111.4	C33—Fe2—C22	82.09 (8)
N2—C19—H19B	111.4	C24—Fe2—C22	63.20 (7)
C20—C19—H19B	111.4	C20—Fe2—C22	63.10 (7)
H19A—C19—H19B	109.3	C23—Fe2—C22	38.45 (7)
C24—C20—C21	110.02 (15)	C21—Fe2—C22	38.40 (6)
C24—C20—C19	108.84 (15)	C1—N1—C9	111.42 (14)
C21—C20—C19	140.99 (17)	C1—N1—C3	113.81 (14)
C24—C20—Fe2	69.96 (10)	C9—N1—C3	107.12 (14)
C21—C20—Fe2	72.94 (10)	C2—N2—C19	112.58 (14)
C19—C20—Fe2	124.93 (13)	C2—N2—C25	113.92 (14)
C20—C21—C22	105.71 (15)	C19—N2—C25	107.39 (14)
C20—C21—Si3	130.20 (14)	C11—Si1—C12	110.03 (11)
C22—C21—Si3	122.23 (13)	C11—Si1—C10	110.07 (12)
C20—C21—Fe2	67.54 (10)	C12—Si1—C10	111.00 (12)
C22—C21—Fe2	78.31 (11)	C11—Si1—C5	109.10 (10)
Si3—C21—Fe2	131.21 (10)	C12—Si1—C5	109.03 (10)
O5—C22—C21	126.57 (18)	C10—Si1—C5	107.54 (10)
O5—C22—C23	126.62 (17)	C15—Si2—C13	110.48 (10)
C21—C22—C23	106.43 (15)	C15—Si2—C14	110.67 (13)
O5—C22—Fe2	133.44 (14)	C13—Si2—C14	110.31 (13)
C21—C22—Fe2	63.29 (10)	C15—Si2—C7	109.32 (11)
C23—C22—Fe2	63.13 (10)	C13—Si2—C7	109.08 (10)
C24—C23—C22	105.65 (15)	C14—Si2—C7	106.89 (10)
C24—C23—Si4	130.75 (14)	C26—Si3—C28	108.84 (11)
C22—C23—Si4	121.98 (13)	C26—Si3—C27	110.21 (12)
C24—C23—Fe2	67.55 (10)	C28—Si3—C27	112.77 (11)
C22—C23—Fe2	78.43 (10)	C26—Si3—C21	108.94 (10)
Si4—C23—Fe2	130.34 (10)	C28—Si3—C21	109.15 (9)
C20—C24—C23	109.83 (16)	C27—Si3—C21	106.86 (9)
C20—C24—C25	108.88 (15)	C30—Si4—C29	110.60 (11)
C23—C24—C25	141.13 (17)	C30—Si4—C31	110.73 (11)
C20—C24—Fe2	70.10 (10)	C29—Si4—C31	110.26 (12)
C23—C24—Fe2	72.77 (10)	C30—Si4—C23	109.75 (10)
C25—C24—Fe2	124.90 (13)	C29—Si4—C23	107.23 (10)
N2—C25—C24	101.70 (14)	C31—Si4—C23	108.17 (10)
